# Teneurin-2 presence in rat and human odontoblasts

**DOI:** 10.1371/journal.pone.0184794

**Published:** 2017-09-19

**Authors:** K. R. Torres-da-Silva, G. W. L. Tessarin, C. A. Dias, I. Z. Guiati, E. Ervolino, A. Gonçalves, I. M. Beneti, D. A. Lovejoy, C. A. Casatti

**Affiliations:** 1 Institute of Biosciences of Botucatu, São Paulo State University, Botucatu, São Paulo, Brazil; 2 Basic Sciences Department, School of Dentistry of Araçatuba, São Paulo State University, Araçatuba, São Paulo, Brazil; 3 Restorative Dentistry Department, School of Dentistry of Araçatuba, São Paulo State University, Araçatuba, São Paulo, Brazil; 4 Department of Surgery and Integrated Clinic, School of Dentistry of Araçatuba, São Paulo State University, Araçatuba, São Paulo, Brazil; 5 Cell and Systems Biology Department, University of Toronto, Toronto, Ontario, Canada; Università degli Studi della Campania "Luigi Vanvitelli", ITALY

## Abstract

Teneurins are transmembrane proteins consisting of four paralogues (Ten-1-4), notably expressed in the central nervous system during development. All teneurins contain a bioactive peptide in their carboxyl terminal named teneurin C-terminal associated peptide (TCAP). The present study analyzed the detailed distribution of teneurin-2-like immunoreactive (Ten-2-LI) cells in developing and mature rat molar teeth, as well as in mature human dental pulps. Ten-2 and TCAP-2 genic expressions were also evaluated in rat and human dental pulps. Finally, Ten-2-LI cells were analyzed during the repair process after dentin-pulp complex injury in rat lower molar teeth. For this, histological sections of rat molar teeth and human dental pulps were submitted to immunohistochemical techniques, while total RNA from developing rat teeth and mature human dental pulps were submitted to conventional RT-PCR. Ten-2-LI cells were evident in the initial bell stage of rat molar teeth development, especially in ectomesenchymal cells of the dental papilla. Ten-2-LI odontoblasts showed strong immunoreactivity in rat and human mature teeth. Ten-2 and TCAP-2 genic expressions were confirmed in rat and human dental pulps. Dentin-pulp complex injury resulted in a decrease of Ten-2-LI odontoblasts after traumatic injury. Interestingly, Ten-2-LI cells were also evident in the pulp cell-rich zone in all postoperative days. In conclusion, Ten-2-LI presence in rat and human odontoblasts was demonstrated for the first time and Ten-2/TCAP-2 genic expressions were confirmed in rat and human dental pulps. Furthermore, it was revealed that Ten-2-LI rat odontoblasts can be modulated during the regenerative process.

## Introduction

Teneurins represent a type II transmembrane glycoprotein with approximately 2800 amino acids, composed of four paralogues (Ten-1-4) and consisting of a number of splice variants in vertebrates [1,2]. This protein was initially isolated and characterized in *Drosophila melanogaster* as a tenascin-like molecule accessory (ten-a) during studies in search of orthologous tenascins (TN), a family of cell-adhesion molecules from the extracellular matrix [1,2]. Subsequently, teneurins were characterized in several invertebrates (tenascin-like molecule major, ten-m) and vertebrates (odz; Ten1-4) showing considerable structural conservation among species [1,2]. Teneurins are mainly expressed during development of the central nervous system (CNS) in rodents and chicken, their expression pattern persists in certain regions during adulthood. Moreover, teneurins are involved with neuronal migration, axonal guidance and neuronal interaction in the CNS [3]. The carboxyl terminal of the teneurins contain a bioactive peptide sequence (40–41 amino acids) with structural similarity to the corticotrophin releasing factor (CRF) named teneurin C-terminal associated peptides (TCAP), related to stress modulation, neuroprotection, among other functions [4–8].

Although the CNS is the major site of teneurin expression, studies show its presence in the orofacial and cervical regions, as well as in other parts of the body, mainly during morphogenesis [9–13]. Functional analysis showed that Ten-1 mutations induced in *Caenorhabditis elegans* resulted in pharyngeal defects [13]. Ten-2 expression was found in the mouse pharyngeal arch mesenchyme [10]; while Ten-3 was also expressed in pharyngeal arches of zebrafish, in condylar cartilage and craniofacial mesenchyme during mouse development [9,11,13,14]. Furthermore, Ten-4 expression was observed in the ectoderm of pharyngeal clefts of chicken, gastrulation stage and mesoderm- and neural-derived tissue of the mouse [15,16]. To corroborate the possible involvement of teneurins in orofacial and cervical development, genetic analyses showed that translocations and trisomies involving 5q34, the locus of the Ten-2 gene, result in craniofacial and limb abnormalities associated with mental retardation [2]. Finally, gene expression analysis showed that Ten-2 (odz-2) was one of 55 genes up-regulated at least four-fold in the dental follicle when compared with the periodontal ligament in human samples [17].

Preliminary screening in our laboratory on teneurin immunoreactivities during rat orofacial development showed that odontoblasts exhibit consistent immunoreactivity to Ten-2. Thus, the present study focused mainly on the presence of teneurin-2-like immunoreactive (Ten-2-LI) odontoblasts by immunohistochemical techniques during development and mature rat molar teeth, as well as in mature human dental pulps. Ten-2 and TCAP-2 genic expressions were also evaluated in rat and human dental pulps by conventional RT-PCR technique. Finally, Ten-2-LI cells were analyzed in rat molar teeth after experimental dentin-pulp complex injury.

## Material and methods

### Animal and human committee approval

The experimental protocols to animal handling and care were approved by the Institutional Committee (FOA—UNESP, process number 2012–02401). Adult male (n = 15) or female (n = 15) Wistar rats were supplied by the central animal house of the School of Dentistry of Araçatuba (UNESP, SP, Brazil).

The experimental protocol using human pulp was approved by the Institutional Human Research Ethics Board (FOA—UNESP, process number 2015–12267). A consent form explaining the detailed purpose of the research project was signed by adult patients (n = 4). Upper and lower third molar teeth (n = 13; 3 or 4 teeth per patient) were extracted due to orthodontic or surgical indications.

### Study delineation

The present study used rat and human samples submitted to different methods and analysis. Table 1 shows a summary of all experimental procedures.

**Table 1:** Summary of experimental delineation using animal and human dental pulp samples.

Species:	Rat (*Rattus novergicus*)		Human dental pulp
Purpose:	Ten-2 and TCAP-2 in tooth development	Ten-2 in dental-pulp complex injury	Ten-2 and TCAP-2 in mature teeth
**Sampling:**	1^st^ and 2^nd^ molar teeth (E-15 to P7)	1^st^, 2^nd^ and 3^rd^ lower molar teeth	3^rd^ molar teeth (dental pulp fragments)
**Method and analysis:**	Indirect Immunofluorescence (Ten-2) and confocal microscope analysis	Superficial occlusal wear	Indirect immunoperoxdase (Ten-2) and light microscope analysis
	Conventional RT-PCR (Ten-2 and TCAP-2) and agarose gel analysis	Sacrificed at 3, 7 and 14 post-operative days	Conventional RT-PCR (Ten-2 and TCAP-2) and agarose gel analysis
		Indirect immunoperoxidase (Ten-2) and light microscope analysis	
**Control reaction:**	Primary antibody omission and adsorption test	Primary antibody omission and adsorption test	Primary antibody omission and adsorption test
	PCR, RT-PCR without template, RT-PCR product subcloning and sequencing		PCR, RT-PCR without template, RT-PCR product subcloning and sequencing

Abbreviations: PCR, polymerase chain reaction; RT-PCR, reverse transcription polymerase chain reaction; TCAP-2, teneurin C-terminal associated peptide-2; Ten-2, teneurin-2.

### Animal

#### Rat tooth development

The rats were divided into cages (3 animals per cage) and kept in the experimental room of the Morphology division of the Department of Basic Sciences for 2 weeks for environmental adaptation. The animals were also maintained in a 12/12 dark-light cycle (lights on 6:00–18:00 h) under constant temperature (22±1°C) and humid (50–60%) conditions, with free access to food and water.

For the tooth development analysis, 3 groups of 4 adult female rats were divided into cages with one adult male rat overnight. In the following morning, the female rats were isolated and those with presence of spermatozoa in the vaginal plug, observed through microscopic examination, were considered to be on day 0 of gestation. Female rats at 15 (E15), 17 (E17), 20 days (E20) of gestation were anesthetized with a lethal dose of sodium pentobarbital and the fetuses were immediately removed by cesarean section. The heads of the fetuses were dissected and immersed in fixative solution containing 4% formaldehyde (Sigma-Aldrich, MO, USA) in 0.1 M sodium phosphate buffer (PBS, Sigma-Aldrich, MO, USA), pH 7.4 overnight (O/N). Newborn male rats, at postnatal day 0 (P0), 5 (P5) and 7 (P7) were deeply anesthetized with ketamine (80 mg/Kg Virbac, SP, Brazil) and xylazine (5 mg/Kg, Bayer, SP, Brazil) and submitted to transcardiac perfusion, initially using 10–20 ml of heparinized saline solution, followed by fixative solution (50–70 ml), as previously described. The heads were dissected and post-fixed O/N. The specimens were decalcified in 10% EDTA (Sigma-Aldrich, MO, USA) in 0.1 M PBS, pH 7.4 for 1–2 weeks at room temperature (RT). The heads were submitted to histological routine processing and paraffin embedding.

In some fetuses (E20), the developing first molar teeth were dissected, immediately immersed in liquid nitrogen and stored at -80°C in ultralow freezer for 1 week, for later RNA extraction.

#### Dentin-pulp complex injury

Adult male rats (n = 9) were divided into cages (three animals per cage) and submitted to dentin-pulp complex injury. For this, the animals were anesthetized as previously mentioned, and superficial wear with different depths (0.2 to 0.5 mm) were created on the occlusal surface of the left lower molar teeth without coronal pulp exposure to the buccal environment, using a spherical diamond bur coupled to a dental high-speed handpiece. Additional animals (n = 3) without any surgical procedures were used as control. The animals were sacrificed at 3, 7 and 14 days after tissue injury by transcardiac perfusion using the same fixative solution, as previously mentioned. The mandibles were dissected and post-fixed overnight. Subsequently, the specimens were decalcified in 10% EDTA in 0.1 M PBS, pH 7.4 for 3 weeks at RT. The mandibles were processed for paraffin embedding.

#### Immunohistochemistry methods

Histological paraffin sections (5 μm thickness) from the coronal plane of heads of fetuses and newborn rats, as well as from the sagittal plane of rat mandibles with dental tissue injury were obtained using a rotary microtome (RM2155, Leica Microsystems, BD, Germany) and collected on positively charged glass slides (Knittel adhesive slides, NS, Germany). The histological sections were dried at RT, kept at 37°C for 2 days in laboratory incubator and at 57°C for 1 hour. The histological sections were deparaffinized in xylenes, and then dehydrated in descending grades of alcohol to distilled water.

For the indirect immunoperoxidase method, sections were washed in 0.1M PBS, pH 7.4 and submitted to antigen retrieval using sodium citrate buffer (10mM sodium citrate, 0.05 Tween 20, pH 6.0, Sigma-Aldrich, MO, USA,) under heat and humid pressure (Decloaking chamber, Model DC2002, Biocare Medical, CA, USA) at 95°C for 5 min. The sections were cooled at RT, submitted to peroxidase endogenous inhibition using 3% hydrogen peroxide (Sigma-Aldrich, MO, USA) in PBS, washed in PBS several times and blocked using 5% non-fat milk diluted in PBS at RT for 1 hour. Additional incubation with 3% bovine serum albumin (BSA, Sigma-Aldrich, MO, USA) in PBS/0.3% Triton X-100 (Sigma-Aldrich, MO, USA) was done at RT for 24 hours to block non-specific antigenic sites. Next, the sections were incubated with primary antibody anti-Ten-2 (1:100, sc-165674, N-13, Santa Cruz Biotechnology, CA, USA) diluted in 3% BSA in PBS/0.3% Triton X-100 at RT for 24 hours. Subsequently, the sections were incubated with secondary biotinylated antibody (1:800, Santa Cruz Biotechnology, CA, USA) and avidin-biotin complex (1:500, ABC, Vector Laboratories, CA, USA) diluted in PBS/0.3% Triton X-100 at RT for 1 hour each step. The immunoreaction was then developed using 0.05% diaminobenzidine chromogen (DAB, Sigma Chemical, MO, USA) and 0.03% hydrogen peroxidase diluted in PBS, under light microscope analysis for reaction control. Finally, the sections were counterstained with hematoxylin (Merck & Co., Inc., NJ, USA), dehydrated, cleared in xylenes and protected using coverslip and DPX mounting medium (Merck & Co., Inc., NJ, USA).

For the indirect immunofluorescence method, sections were submitted to antigen retrieval, non-specific blocking using 5% non-fat milk, followed by secondary blocking using 3% BSA in PBS/0.3% Triton X-100 and incubated with Ten-2 primary antibody. Next, sections were incubated with specific secondary antibody, followed by streptavidin conjugated with Cy^3^ (1:500, Jackson Immunoresearch, PA, USA) and counterstained with DAPI nucleus staining (Biosensis, SA, Australia). The sections were protected with glycerol mounting medium and coverslips.

Immunohistochemical control reactions were performed using primary antibody omission or adsorption test using Ten-2 primary antibody (Ten-2, 1:100, sc-165674, N-13, Santa Cruz Biotechnology, CA, USA) at different antibody/peptide (1:1; 1:0.1; 1:0.01; 1:0.001) concentrations (Ten-2, sc-165674p, N-13, 100 μg/0.5 ml, Santa Cruz Biotechnology, CA, USA).

Histological sections from developing rat molar teeth submitted to indirect immunofluorescence method were qualitatively analyzed to identify Ten-2-LI cells, using standardized excitation and emission filters for visualization of DAPI (nuclear staining) and Cy^3^ (streptavidin-conjugated to identify Ten-2 antibody), in a confocal microscope (Leica DMI 6000CS, GmbH, Germany) equipped with diode, helium-neon and argon lasers (TCS-SP5 model, AOBS Tandem Scanner, Leica, GmbH, Germany). The histological sections were evaluated using 20x, 40x and 63x plan apochromatic objectives, and selected areas were captured (0.5 μm increments) in TIFF formats. The digital images were adjusted for brightness, contrast and intensity, without changing the immunolabeling pattern using Corel Draw software.

Histological sections of rat lower molar teeth with tissue injury or from control animals, submitted to indirect immunoperoxidase, were qualitatively analyzed to identify Ten-2-LI cells using a light microscope (Axiolab A1, Carl Zeiss, Göttingen, Germany) coupled to a digital camera (AxioCam MRc5, Carl Zeiss, Göttingen, Germany). The selected areas were captured using imaging software (Zen2, Carl Zeiss, Göttingen, Germany). Brightness, contrast and intensity were adjusted in all digital images, as previously mentioned.

#### Reverse transcriptase PCR

Developing rat molars (crown formation stage) stored at -80°C in ultralow freezer were thawed and coronal pulp samples were collected using a curette and tweezers under surgical stereomicroscopy (Model MC A-199, DF Vasconcellos, SP, Brazil). These dental pulp fragments were transferred to appropriate centrifuge tubes containing 1.0 ml trizol (Life Technologies, CA, USA) and immediately homogenized (Tissue-Tearor, Biospec Products, CA, USA) at 30000 rpm for 40 sec, incubated at RT for 5 min and centrifuged at 12000 g for 5 min. The supernatant was transferred to a new microcentrifuge tube and 200 μl of chloroform (Biochemicals Life Science Research Products, OH, USA) was added, vigorously vortexed for 15 sec and incubated on ice during 3 min. The solution was submitted to refrigerated centrifugation at 12000 g, 4°C during 15 min and the upper phase containing the total RNA was transferred to a new microcentrifuge tube. Subsequently, 0.5 ml of isopropyl alcohol (Sigma Chemical, MO, USA) was added to the total RNA solution, incubated at RT for 10 min and centrifuged at 12000 g, 4°C for 10 min. The supernatant was discarded, 1 ml of 75% alcohol (Sigma Chemical, MO, USA) was added to disrupt the pellet and the solution was centrifuged at 7500 g, 4°C for 5 min. The alcohol solution was carefully removed, the pellet was dehydrated at RT during 5–10 min and then resuspended in 100 μl of sterilized nuclease free water to obtain a final solution with total RNA.

To ensure total RNA concentration without contamination, the previously extracted RNA was submitted to an additional purification process using resin column system (R2052, Direct-zol RNA MiniPrep, Zymo Research, CA, USA) followed by DNAse treatment (AM1907, Turbo DNA-free kit, Ambion, Life Technologies, CA, USA), according to the manufacturer’s instructions. To confirm RNA quantity and quality, the total RNA was measured using a spectrophotometer (Optizen POP nano bio, Mecasys, Daejeon, South Korea) and submitted to electrophoresis on 1.5% denaturing agarose gel.

Conventional RT-PCR was used to amplify rat and human Ten-2, TCAP-2 and beta-actin using a commercial kit (210212, One-step RT-PCR, Qiagen, CA, USA). The RT-PCR solution contained 10 μl OneStep RT-PCR buffer, 2 μl dNTP mix, 1 μl of each primer (10 nM) (Table 2), 2 μl OneStep RT-PCR enzyme mix, 4.0 μl of each RNA (7.2–97.2 ng/μl) and 30 μl RNase-DNase free water. The RT-PCR solution was submitted to cDNA synthesis at 50°C for 30 min, an initial denaturation at 95°C for 15 min, 35–45 amplification cycles (94°C for 1min, 45°-53°C for 1 min and 72°C for 1 min) and final extension at 72°C for 10 min. The RT-PCR products were analyzed by electrophoresis on a 1.5% agarose gel (9012-36-6, Biotechnology Grade, OH, USA) stained with ethidium bromide (1239-45-8, Sigma Aldrich, CA, USA). Control reactions were performed without RNA addition in the RT-PCR or with RNA addition in the PCR assays (C1141, GoTaq Flexi DNA Polymerase, Promega, WI, USA). Agarose gel images were captured using ImageQuant LAS 500 (GE Healthcare Bio-sciences, Uppsala, Sweden) and electronic files were qualitatively analyzed using ImageQuant Tl software (GE Healthcare, Bio-sciences, Uppsala, Sweden).

**Table 2:** List of primers used in conventional RT-PCR for rat and human samples.

Gene	Forward primer	Reverse primer	Exon	Size (bp)	Access number
Rat Ten-2	5'-tgtgactgcaaaaacgatgtcaac-3'	5'-tcccatcataagtcatgaggcccagc-3’	23	495bp	NM011856.2
Rat TCAP-2	5'-gacaagatgcactacagcatcgag-3'	5'-ccatctcattctgtcttaagaactgg-3'	28	496bp	NM011856.2
Rat β-actin	5'-caggtcatcactattggcaacgag-3'	5'-ctcatcgtactcctgcttgctgat-3'	4–6	357bp	NM007393
Human Ten-2	5'-gagaacaatgtcatccttcgaatc-3'	5'-cgttgaaaacatataactcctgctc-3'	23	495bp	NM1122679
Human TCAP-2	5'-gacaagatgcactacagcatcgac-3'	5'-ccatctcattctgtcttaaaaactgg-3'	29	496bp	NM1122679
Human β-actin	5'-catgtacgttgctatccaggc3'	5'-ctccttaatgtcagccacgat-3'	4	250bp	NM001101

In order to confirm that the RT-PCR products were from Ten-2 or TCAP-2 mRNA amplification, some samples were purified from gel (A9282, Wizard SV gel and PCR Clean-Up System, Promega, WI, USA) and subcloned in plasmids (A1380, pGEM-T Easy Vector System, Promega, WI, USA). The bacteria carrying plasmids with positive clones were selected using blue-white colony screening and the plasmids were purified (A1223, PureYield Plasmid Miniprep System, WI, USA). Plasmids were submitted to restriction digestion to confirm the subcloned fragment sizes and then commercially sequenced. The amplified sequences were analyzed by similarity sequence using public bioinformatics software (Blast sequence similarity search, https://blast.ncbi.nlm.nih.gov/Blast.cgi).

### Human dental pulp

Patients (n = 4), previously selected by a dentist from the School of Dentistry of Araçatuba (São Paulo State University, Araçatuba, SP, Brazil), were submitted to extraction of erupted or partially erupted third molar teeth. Three or four third molar teeth were extracted from each patient after buccal antisepsis using 0.12% chlorhexidine solution (Periogard, Colgate-Palmolive, SP, Brazil), local anesthesia with topic anesthetic gel, and regional and local anesthesia using 2% mepivacaine (DFL, RJ, Brazil). Two third molar teeth (from each patient) were cleaned in 0.12% chlorhexidine solution (Periogard, Colgate-Palmolive, SP, Brazil), longitudinally fractured using forceps, immediately immersed in liquid nitrogen, transferred and stored at -80°C in ultralow freezer for one week, for later RNA extraction. The remaining teeth (1 or 2 third molar teeth of each patient) were similarly extracted and fractured and the specimens were then immediately immersed in fixative solution O/N. Next, the fractured teeth were dissected to collect coronal pulp fragments using a curette and tweezers under surgical stereomicroscopy (Model MC A-199, DF Vasconcellos, SP, Brazil). The dental pulp fragments were submitted to paraffin embedding.

#### Immunohistochemistry methods

Histological sections of human coronal pulp were cut at 5 μm thickness in a rotary microtome (RM2155, Leica Microsystems, BD, Germany) and collected in positively charged glass slides (Knittel adhesive slides, NS, Germany). The histological sections were treated with the indirect immunoperoxidase method and subsequently qualitatively analyzed to identify Ten-2-LI cells using a light microscope, as previously mentioned. Immunohistochemical control reactions were performed by primary antibody omission or adsorption test as previously described.

#### Reverse transcriptase PCR

Fractured third molars stored at -80°C in ultralow freezer were thawed and coronal pulp samples were collected using a curette and tweezers under surgical stereomicroscopy (Model MC A-199, DF Vasconcellos, SP, Brazil). These dental pulp fragments were submitted to total RNA extraction, RT-PCR and electrophoresis on 1.5% denaturing agarose gel. Control reactions were also performed as previously described.

## Results

### Rat molar tooth development

**Fig 1:**
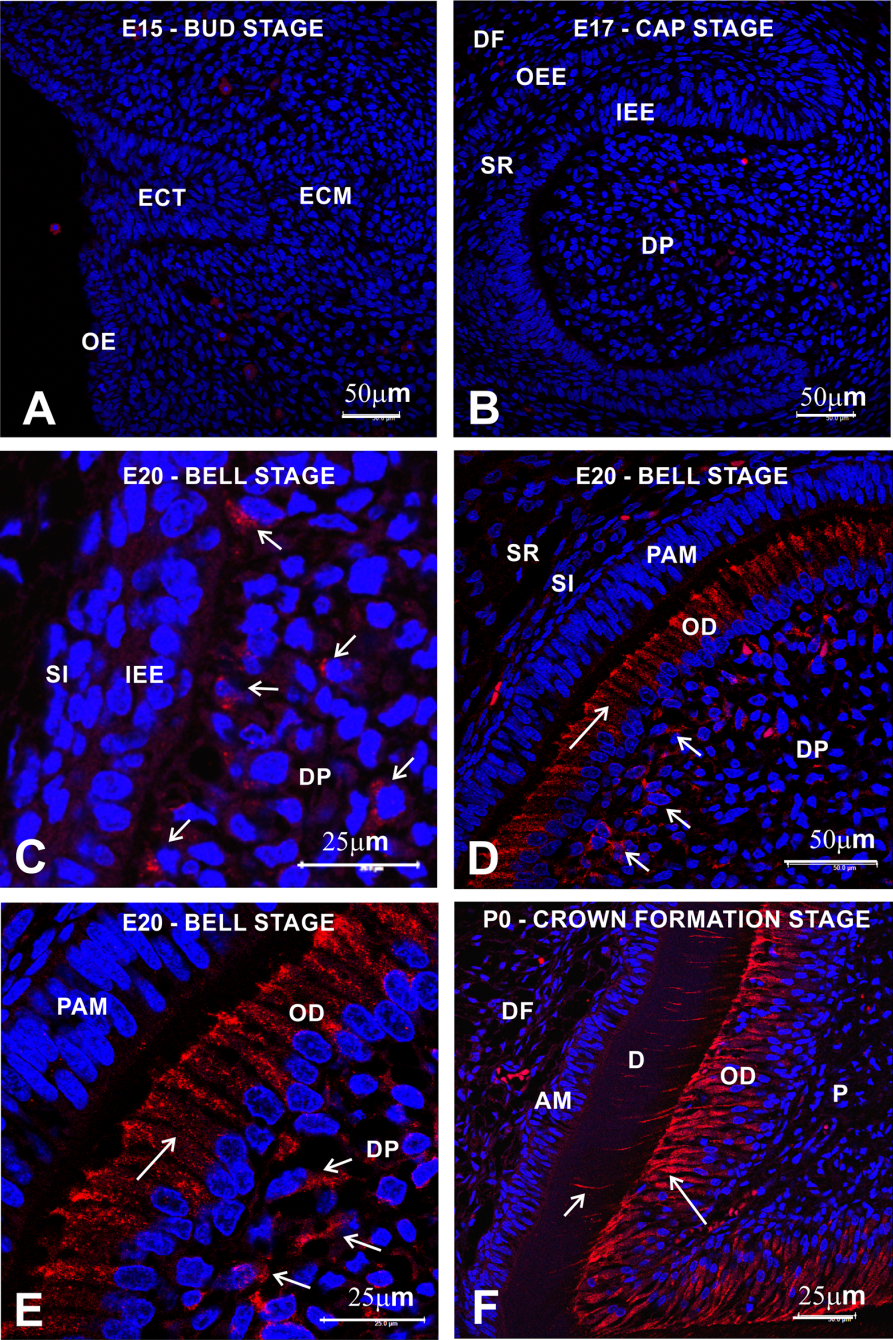
Ten-2-LI immunofluorescence analysis during rat tooth development. Confocal microscope photomicrographs showing histological sections of sequential development of rat molar teeth (A, bud stage; B, cap stage; C, initial bell stage; D-E, advanced bell stage; F, crown formation). Immunoreactivity to Ten-2 is only evident in ectomesenchymal cells (small arrows) in peripheral region of dental papilla in initial bell stage of tooth development (C) and in newly differentiated odontoblasts during advanced bell stage (large arrow) (D, high magnification is shown in E). In F, mature odontoblasts producing dentin organic matrix, exhibiting intense immunoreactivity to Ten-2 in cell body (large arrow) and in their cell process inside dentinal tubules (small arrow) during crown formation. Abbreviations: AM, ameloblasts; D, dentin; DF, dental follicle; DP, dental pulp; ECT, ectoderm layer; ECM, ectomesenchyme; IEE, inner enamel epithelium; OD, odontoblasts; OE, oral epithelium; OEE, outer enamel epithelium; P, dental pulp; PAM, pre-ameloblasts; SI, stratum intermedium; SR, stellate reticulum.

Indirect immunofluorescence technique was used to identify Ten-2 like-immunoreactive (Ten-2-LI) cells in the lower first and second molar teeth during development. E15 embryos had molar teeth in the bud and cap stages and no immunolabeling was detected (Fig 1A and 1B). However, Ten-2-LI presence was initially evident in rat ectomesenchymal cells of the dental papilla subjacent to the inner enamel epithelium layer during the initial bell stage in E20 embryos (Fig 1C). Ten-2 immunoreactivity significantly increased in the late bell stage, specifically in the odontoblast cell layer positioned close to pre-ameloblasts in E20 embryos (Fig 1D and 1E). In addition, Ten-2 immunoreactivity was present in ectomesenchymal cells, subjacent to the odontoblast cell layer in the dental papilla in E20 embryos (Fig 1D and 1E). Interestingly, Ten-2 immunoreactivity was present in the cell cytosol (Fig 1D and 1E). This immunoreactivity remained significant in odontoblasts during crown formation in P0-P7 newborn rats (Fig 1F).

**Fig 2:**
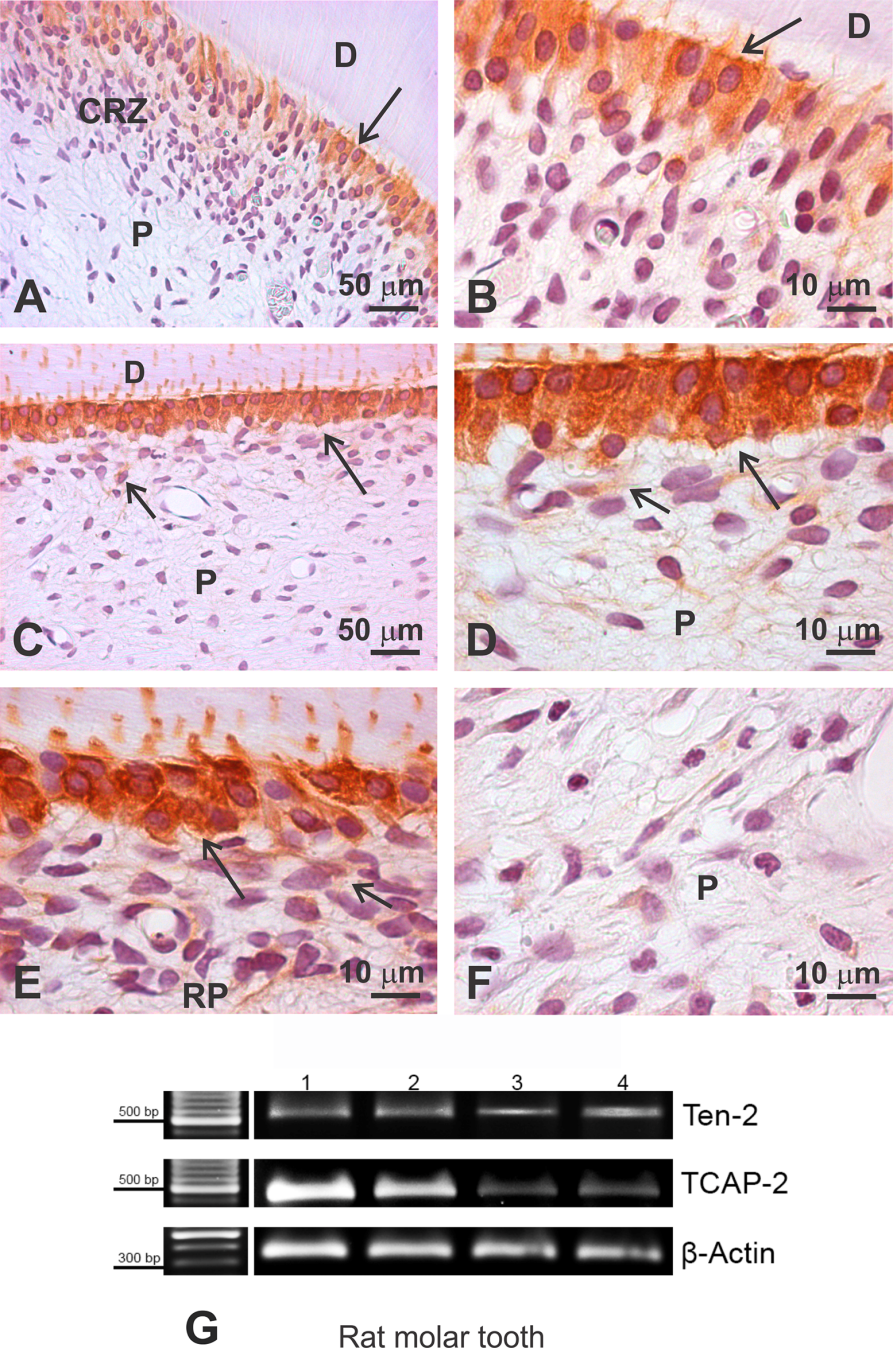
Ten-2-LI immunoperoxidase analysis in dental pulp of rat mature molar tooth and conventional RT-PCR. Note presence of intense immunolabeling to Ten-2 in odontoblasts (large arrow), positioned in roof of coronal pulp (A, high magnification in B), lateral wall (C, high magnification in D) and in radicular pulp (E). In F, observe presence of discreet immunolabeled cells subjacent to odontoblast layer (small arrow) and no immunoreactive cells in center of dental pulp. In G, expression of Teneurin-2 and TCAP-2 in 1.5% agarose gel using total RNA extracted from rat molar pulp during crown formation. Abbreviations: CRZ, cell-rich zone; D, dentin; P, dental pulp; RP, radicular pulp; TCAP-2, teneurin C-terminal associated peptide-2; Ten-2, teneurin 2.

In mature teeth, Ten-2 immunoreactivity also remained intense and diffusely distributed in the cell bodies and processes of odontoblasts positioned in the coronal and radicular pulps (Fig 2A–2E). Occasionally, Ten-2-LI cells exhibiting discreet immunoreactivity were also evident subjacent to the odontoblast layer (Fig 2C–2E), whereas Ten-2-LI cells were not observed in other dental (Fig 2F) or periodontal cell types. Conventional RT-PCR confirmed Ten-2 and TCAP-2 expressions in developing rat molar teeth (Fig 2G).

Ten-2 control reactions, by omission of primary antibody or adsorption tests, showed no immunolabeling in the histological sections of developing or mature teeth (S1 Fig). The RT-PCR (without RNA) or PCR control reactions showed no Ten-2 or TCAP-2 expressions in rat samples, confirming absence of DNA contamination in the samples. Sequencing analysis from plasmids subcloned with RT-PCR products confirmed the identity of Ten-2 and TCAP-2 amplified products.

### Rat dentin-pulp complex injury

In order to analyze the dental pulp cell regeneration response during tissue injury, superficial occlusal wear was performed at different degrees in the lower molars to induce different inflammation grades, and the dental pulp region was evaluated at 3, 7 and 14 postoperative days.

**Fig 3:**
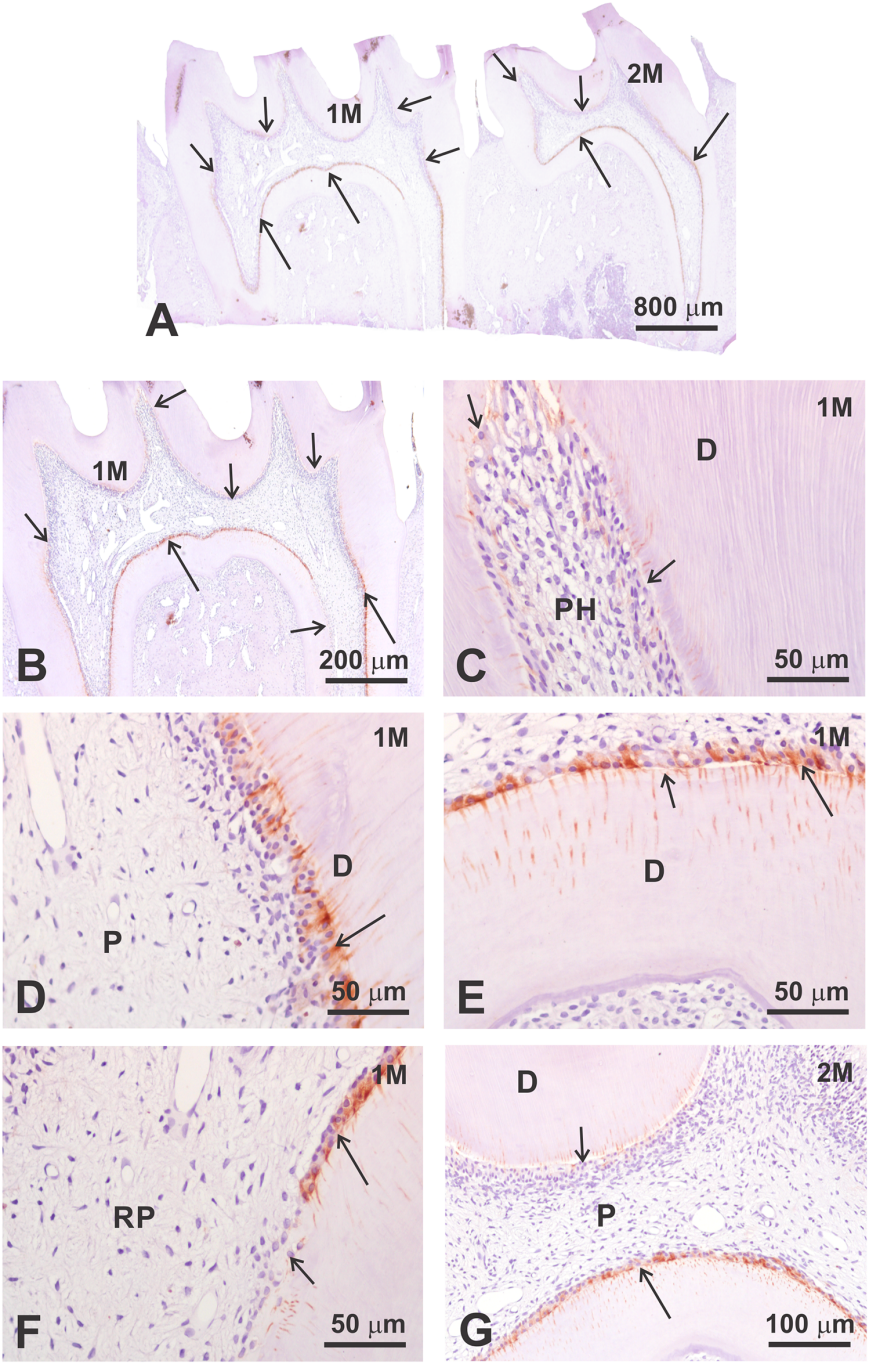
Ten-2-LI immunoperoxidase analysis in dental pulp of rat mature molar tooth three days after slight dentin-pulp complex injury. Note that this traumatic injury did not induce significant inflammation process in dental pulp. However, it was enough to significantly decrease immunoreactivity to Ten-2 in odontoblasts mainly in pulp horn, roof and lateral walls of coronal pulp (small arrow). Some immunolabeling persisted in some odontoblasts present in floor of coronal pulp and radicular pulp (large arrow). Abbreviations: 1M, lower first molar tooth; 2M, lower second molar tooth; P, dental pulp; PH, pulp horn; D, dentin; RP, radicular pulp.

In mild inflammation induced in the coronal pulp, only a discreet pulp tissue change was evident after 3 postoperative days (Fig 3A–3G). In this case, Ten-2 immunoreactivity was significantly downregulated only in odontoblasts positioned in the pulp horn (Fig 3A–3G), roof and lateral walls of the coronal pulp, whereas odontoblasts in the floor and in the radicular pulp showed significantly decreased immunolabeling (Fig 3D–3G).

**Fig 4:**
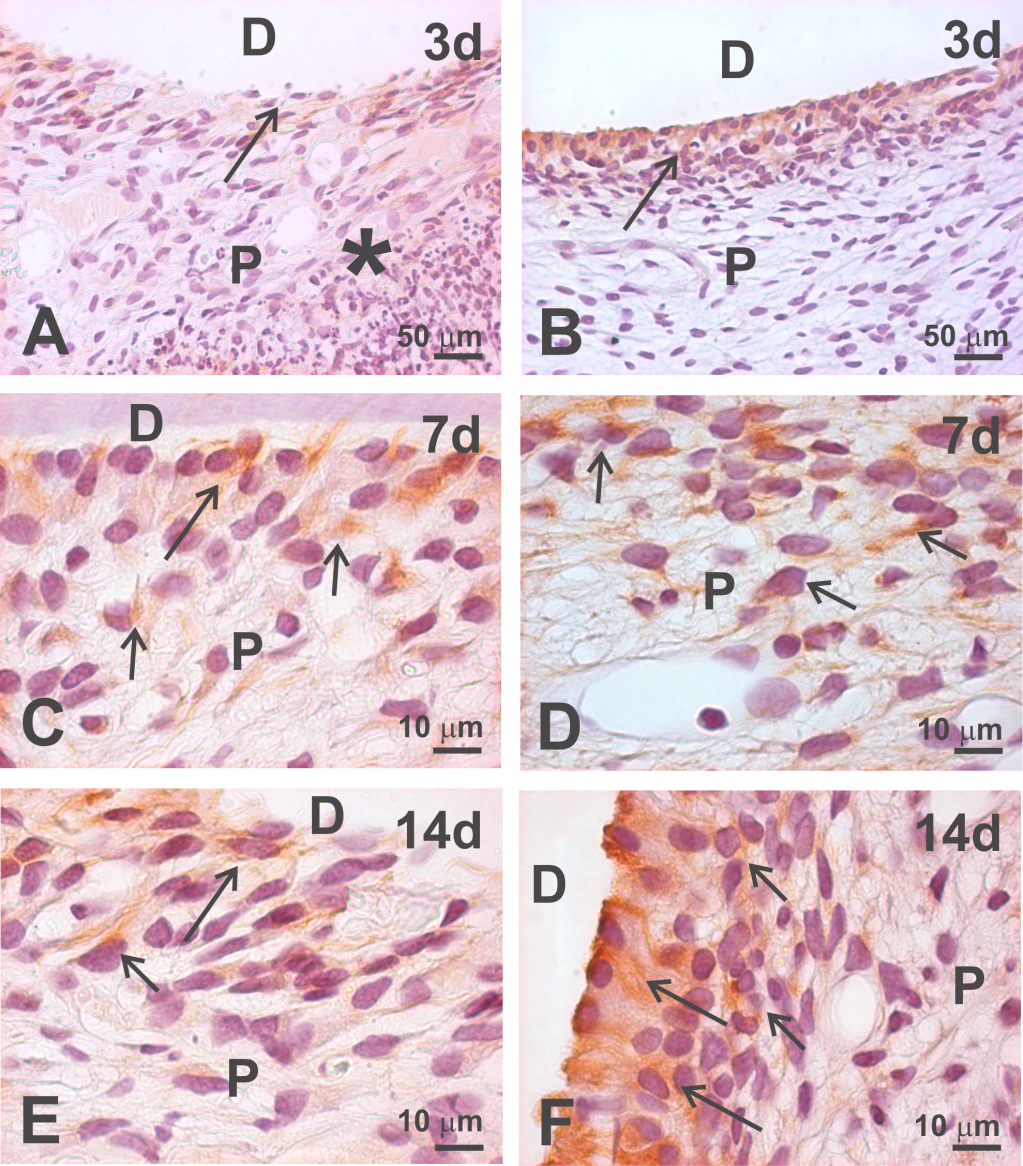
Ten-2-LI immunoperoxidase analysis in dental pulp of rat mature molar tooth after moderate and severe dentin-pulp complex injury. In A-B, presence of some odontoblasts or odontoblast-like cells (large arrow) exhibiting discreet immunolabeling to Ten-2, after three postoperative days (3d). In C, it is possible to identify some odontoblasts (large arrow) and subjacent cells (small arrows) exhibiting discreet immunolabeling to Ten-2. D shows Ten-2-LI cells (small arrows) in the cell-rich zone after 7 postoperative days (7d). E shows a still disorganized odontoblast layer with discreet immunolabeling in odontoblast-like cells (large arrow) and in subjacent cells (small arrow), after 14 postoperative days (14d). F shows a more organized odontoblast layer with odontoblasts (large arrows) exhibiting moderate immunolabeling to Ten-2 after 14d. Abbreviations: *, inflammatory process; P, dental pulp; D, dentin.

In other experimental cases with induction of moderate or severe inflammation in the dental pulp, Ten-2 immunoreactivity was completely downregulated in all odontoblasts present in the coronal and radicular pulps, with some residual immunolabeling remaining in the odontoblastic process within the dentinal tubules. The inflamed lesion disrupted the typical odontoblastic layer in some cases and the remaining odontoblasts or odontoblast-like cells showed non-homogenous immunolabeling in all postoperative periods (Fig 4A–4F). An important finding was that more cells in the cell-rich zone of the pulp exhibited Ten-2 immunoreactivity (Fig 4A–4F). The odontoblast layer was re-established in the latest postoperative period in some experimental animals, where odontoblasts showed a tendency to increase immunoreactivity to Ten-2 (Fig 4E and 4F).

Although not the primary focus of this study, it is important to mention that clear immunoreactivity to Ten-2 was noticed in cementoblasts around the apical foramen (S2 Fig) and in osteoblasts distributed in the alveolar wall of the cervical region of the interradicular septum and in the periapical region (S2 Fig). This immunolabeling pattern was only observed in animals submitted to dentin-pulp complex injury.

### Human dental pulp

**Fig 5:**
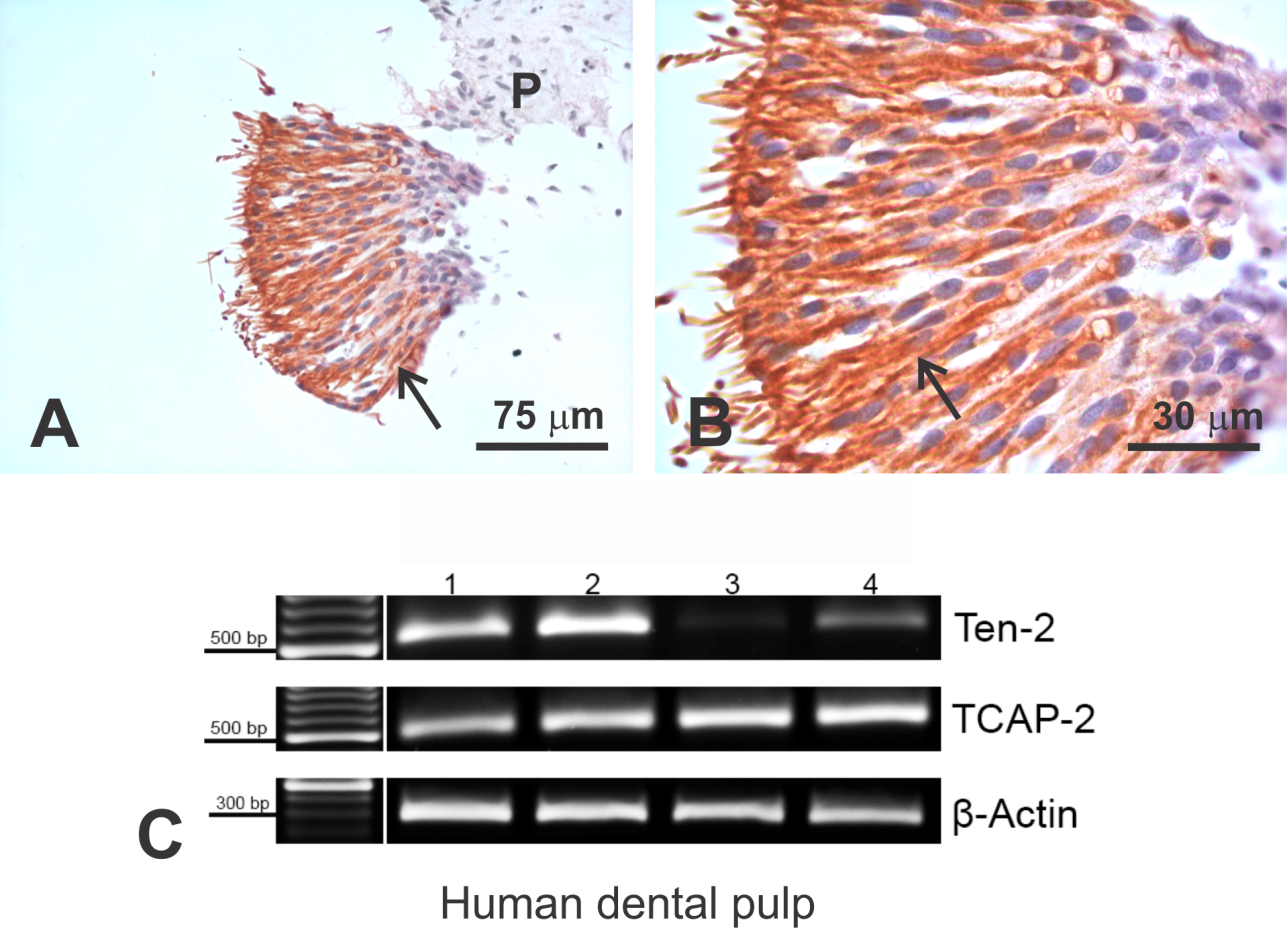
Ten-2-LI immunoperoxidase analysis of human dental pulp and conventional RT-PCR. In A-B, odontoblast layer at high magnification with intense Ten-2-LI (arrow). In C, 1.5% agarose gel stained with bromide ethidium exhibiting Ten-2, TCAP-2 and beta-actin RT-PCR-amplified products using total RNA extracted from human third molar dental pulp. Abbreviations: P, dental pulp; TCAP-2, teneurin C-terminal associated peptide-2; Ten-2, teneurin 2; β-actin, beta actin.

Coronal dental pulp fragments showed clear Ten-2-LI odontoblasts (Fig 5A and 5B). This immunolabeling was homogenously distributed in the cells. No pulp cells, such as fibroblasts or undifferentiated mesenchymal cells exhibited immunolabeling to Ten-2 (Fig 5A and 5B). Sometimes, the initial segment of the odontoblastic process was preserved, showing Ten-2-LI presence, similar to rat odontoblasts (Fig 5A and 5B). Ten-2 control reactions by omission of primary antibody or adsorption test showed no immunolabeling in the histological sections of human pulp (S3 Fig). RT-PCR analysis confirmed Ten-2 and TCAP-2 expressions in human coronal pulp samples (Fig 5C). The RT-PCR (without RNA) or PCR control reactions showed no Ten-2 or TCAP-2 expressions, confirming absence of DNA contamination in the samples. Sequencing analysis from plasmids subcloned with RT-PCR products confirmed Ten-2- and TCAP-2-amplified products.

## Discussion

The present study is the first to demonstrate Ten-2 presence during rat tooth development and in rat or human mature teeth. Both rat and human odontoblast cells exhibited strong immunoreactivity to Ten-2. Ten-2 and TCAP-2 expressions were confirmed in developing rat molar teeth, as well as in mature human dental pulps using RT-PCR. In addition, Ten-2 immunoreactivity was down-modulated in rat odontoblasts after dentin-pulp complex injury.

Teneurins are transmembrane proteins mainly present in the nervous tissue and primarily related to neuronal interaction [1–3]. However, there is increased evidence of their expression in non-neuronal tissues [9–13,15]. Previous studies reported teneurin expression in the viscerocranial region of mouse; however, a possible relation to tooth development was not mentioned [9–12]. An additional study based on gene expression analysis indicated the presence of Ten-2 (odz-2) in the dental follicle of human samples [17]. In the present study, Ten-2 immunoreactivity in the odontoblast cell lineage was observed since its initial differentiation from ectomesenchymal cells. This initial observation suggests that local cell differentiation and/or growth factors are stimulating Ten-2/TCAP-2 in odontoblasts during tooth development.

Mature odontoblasts exhibited elevated immunoreactivity to Ten-2, homogenously distributed in the cell body and in the cell process within the dentinal tubules. Moreover, this immunolabeling pattern was consistently down-regulated in odontoblasts after dentin-pulp complex injury induced in rat molar teeth. Recent studies have shown that teneurins establish homophilic or heterophilic molecular interactions, contributing to cell adhesion in neurons, which is fundamental to circuitry development and synaptic connections [3, 18–20]. It is important to mention that odontoblasts preserve some neural phenotype, as this cell lineage is derived from neural crest cells [21,22]. Thus, Ten-2 may act as a hemophilic cell adhesion molecule in odontoblasts, contributing for these cells to establish intercellular functional coupling. Additionally, Ten-2-LI down regulation in odontoblasts during dental injury can facilitate disruption of the odontoblast layer, facilitating odontoblast migration and reorganization to produce reactional tertiary dentin and protect the dental pulp.

Ten-2 heterophilic interactions have also been raised with latrophilin, integrin and dystroglycan proteins [3,8,23–26]. Latrophilin proteins (LPHN) belong to the adhesion G protein-coupled receptor subfamily L and are constituted of three homologues (LPHN1, LPHN2 and LPHN3) [20,23,26]. A probable heterophilic interaction between Ten-2 and LPHN proteins in odontoblasts needs further analysis, since we were unable to identify convincing LPHN immunoreactivities in odontoblasts of histological sections of rat and human teeth (unpublished data). Integrins are strong candidates for heterophilic interaction with Ten-2 in odontoblasts, as previous studies showed undifferentiated and mature rat and human odontoblasts expressing some integrin isoforms [27,28]. The presence of integrin molecules in the cell-cell adhesion mechanism is important to maintain organization and cohesion of the odontoblast layer [27]. Finally, Ten-2 interaction with dystroglycan proteins is less probable as only α-dystroglycan mRNA expression was observed during tooth development and it is not present in odontoblasts [29].

The Ten-2 immunoreactivity pattern was similar between rat and human odontoblasts, demonstrating that this protein is preserved in this cell lineage throughout mammalian evolution. TCAP-2 immunoreactivity was not evaluated as there is no available specific antibody for this sequence. Concerning the Ten-2 immunoreactivity staining pattern, some points need consideration, given that Ten-2 immunolabeling was homogenously distributed in all cell parts of the odontoblast. This immunolabeling pattern is different from *in vivo* and *in vitro* studies, which showed that teneurin immunoreactivity is mainly present in the neuronal cell membrane [18,19]. The diffuse immunoreactivity to Ten-2 present in the cytoplasm of rat and human odontoblasts can raise a discussion on other possible functions attributed to teneurins in non-neuronal cells. Firstly, it is important to mention that the antibody used in this study recognized the intracellular part of this transmembrane protein, as previously mentioned. Therefore, this antibody might be identifying Ten-2 linked to membrane, as well as intracellular protein parts that are processed and translocated to different parts of the cell, justifying the diffuse immunolabeling pattern observed in odontoblasts. Previous studies have shown that the intracellular part of Ten-1 and Ten-2 can be translocated to the nucleus acting as transcription factors [30,31]. Additionally, teneurins have extracellular cleavage residues [32]. In line with this, it is known that the extracellular part presents two potential cleavage sites. One is a conserved furin cleavage in the extracellular domain, between the transmembrane domain and the first EGF-like repeats that release the large extracellular sequence to the extracellular environment. The other one is a furin cleavage site near the C-terminal that may release TCAP [33]. The fate of the teneurin extracellular fragments and TCAP have been discussed in some studies [32–33]. TCAP-1 acts as a neuroprotective molecule against alkalotic stress, influences brain-derived neurotrophic factor expression (BDNF) in immortalized hypothalamic neurons, as well as increases β-actin and tubulin, modulating neurite outgrowth in primary hippocampal neurons [7,33–35], while Ten-2 fragments can be incorporated to the extracellular matrix [10,18]. Finally, it is important to mention that there are at least four Ten-2 splice variants in humans [32]. The recently characterized Ten-2 splice variant named latrophilin-1-associated synaptic surface organizer (Lasso) generates additional proteins by differential proteolysis, which can act in paracrine cell signaling [23,26]. Taken together, these findings suggest that Ten-2 and its related proteins may be involved in other biological activities in odontoblasts under normal and pathological conditions, besides acting as a transmembrane protein for cell-cell and/or cell-extracellular matrix interactions.

Interestingly, Ten-2 immunoreactivity was detected in cells present in the cell-rich zone of the pulp in all postoperative days after dental injury. The cell-rich zone of the pulp is known as a source-rich region of undifferentiated mesenchymal cells [36,37]. This finding is aligned with our results showing that ectomesenchymal cells from dental papilla exhibited Ten-2 immunoreactivity, prior to odontoblast differentiation. Previous studies showed that teneurins are involved with nervous and muscle tissue regeneration processes [38,39]. Additional data from our laboratory demonstrated Ten-2 presence in astrocytes after mechanical brain lesion, supporting its role during tissue regeneration (submitted manuscript). Thus, Ten-2 up-regulation in undifferentiated mesenchymal cells present in the cell-rich zone of the pulp can be a key mechanism to stimulate odontoblast differentiation during the dentin-pulp complex repair process.

Recent studies have explored the use of small biological molecules as a clinical approach for natural tooth and periodontium repairs, such as agonists or antagonists for intracellular signaling pathways [40,41]. For example, small molecules that up-regulate Wnt/β-cat signaling pathway have been delivered directly in the dental pulp, stimulating differentiation of resident stem cells in odontoblasts; thus, inducing tertiary dentin deposition [40]. Based on the data from the present study and on this new approach, up-regulation of Ten-2/TCAP-2 in the dental pulp can also be an adjuvant treatment for dental trauma or carious lesion, associated with restorative procedures. Considering this possibility, potential Ten-2 inducers may be tested during dental pulp repair in further studies. It is also important to mention that TCAP (40–41 amino acids) is a natural peptide that can be used as an adjuvant treatment, as it acts as a protective molecule against alkalotic stress in neurons, among other beneficial roles [6–8]. In addition, *in vitro* assays demonstrated that TCAP-1 treatment significantly increased Ca^++^ influx in neurons and astrocytes (unpublished data), as well as activated MEK1/2 and ERK1/2 intracellular pathways in hippocampal cells [24]. These TCAP-1 roles may be important in odontoblast differentiation during dental pulp repair. For instance, extracellular Ca^++^ stimulates odontoblast differentiation from human dental pulp stem cells (hDPSCs), upregulating bone morphogenetic protein (BMP)-2 through SMAD1/5/8 and ERK1/2 pathways [42]. In addition, this cell differentiation mechanism is dependent on intracellular calcium signaling [43].

Another important finding was the presence of Ten-2 immunoreactivity in osteoblasts and cementoblasts positioned in specific regions. Immunolabeled osteoblasts were positioned in the alveolar bone wall, particularly in the most cervical part of the inter-radicular septum and around the periapical region. The occlusal surface of the lower molar teeth was worn out in order to induce dentin-pulp injury, keeping them in infraocclusion position. In this hypofunction condition, osteoblasts increase bone production in the alveolar surface, mainly in the periapical region, reducing the periodontium space [44,45]. Thus, it is possible that only significantly activated osteoblasts should be expressing Ten-2. In relation to cementoblasts, only those in the apical region of the root also exhibited immunoreactivity to Ten-2. These data can also indicate that, similar to osteoblasts, only activated cementoblasts exhibited immunolabeling. Previous studies demonstrated that hypofunctional teeth increase cementum deposition, mainly in the apical region of the dental root, in order to induce tooth displacement to the occlusal plane as a compensatory mechanism [44,45]. Additional studies are necessary to confirm the real role of Ten-2 presence in osteoblasts and cementoblasts and whether or not this protein can be used as a potential marker for these activated cells. Similarly, as previously discussed in relation to odontoblasts and dentin repair, Ten-2/TCAP-2 regulation may also be an adjuvant therapy for endodontics (apical foramen sealing after pulpectomy) and surgical (alveolar bone repair) procedures.

In conclusion, the present study demonstrated that immunoreactivity to Ten-2 is present in odontoblasts during differentiation, in mature odontoblasts, as well as during the repair process after induced injury in the dentin-pulp complex. Ten-2 and TCAP-2 gene expressions were also evident in rat teeth and in human dental pulps. Further studies are necessary to elucidate the function of the Ten-2/TCAP-2 system in odontoblasts and explore its potential use as adjuvant therapy in clinical procedures for dental pulp repair.

## Supporting information

S1 Fig Ten-2-LI adsorption test in rat mature molar tooth. Histological section of mature lower molar teeth of rat (A-D) submitted to adsorption test (1:1, Ten-2 peptide/antibody concentrations) and indirect immunoperoxidase method. A-C show sequential resolution of rat second molar tooth evidencing no immunolabeling in odontoblasts (arrows) or in other pulp cells. D shows high magnification of odontoblast layer with absence of immunolabeling (arrows) in radicular pulp. Abbreviations: 2M, lower second molar; D, dentin; RP, radicular pulp.
(TIF)

S2 Fig Ten-2-LI immunoperoxidase analysis in the periodontium of rat mature molar tooth under injury. In A-B, presence of cementoblasts (arrows) around the apical foramen (asterisk) exhibiting strong immunolabeling to Ten-2. C-D show some osteoblasts (arrows) situated in the interradicular septum and in the periapical region exhibiting strong Ten-2 immunoreactivity. Abbreviations: BT, bone trabeculae; C, cementum; D, dentin; RP, radicular pulp.
(TIF)

S3 Fig Ten-2-LI adsorption test in human dental pulp. Histological section of mature human dental pulp submitted to adsorption test (1:1, Ten-2 peptide/antibody concentrations) followed by indirect immunoperoxidase method. In A-B, odontoblast layer at high magnification with absence of immunolabelling (arrow). Abbreviation: P, dental pulp.
(TIF)
