# Supplementary figures and images for: Teneurin-2 presence in rat and human odontoblasts
Source: PLoS One. 2017 Sep 19;12(9):e0184794. doi: 10.1371/journal.pone.0184794 (PMC5604987; doi:10.1371/journal.pone.0184794)

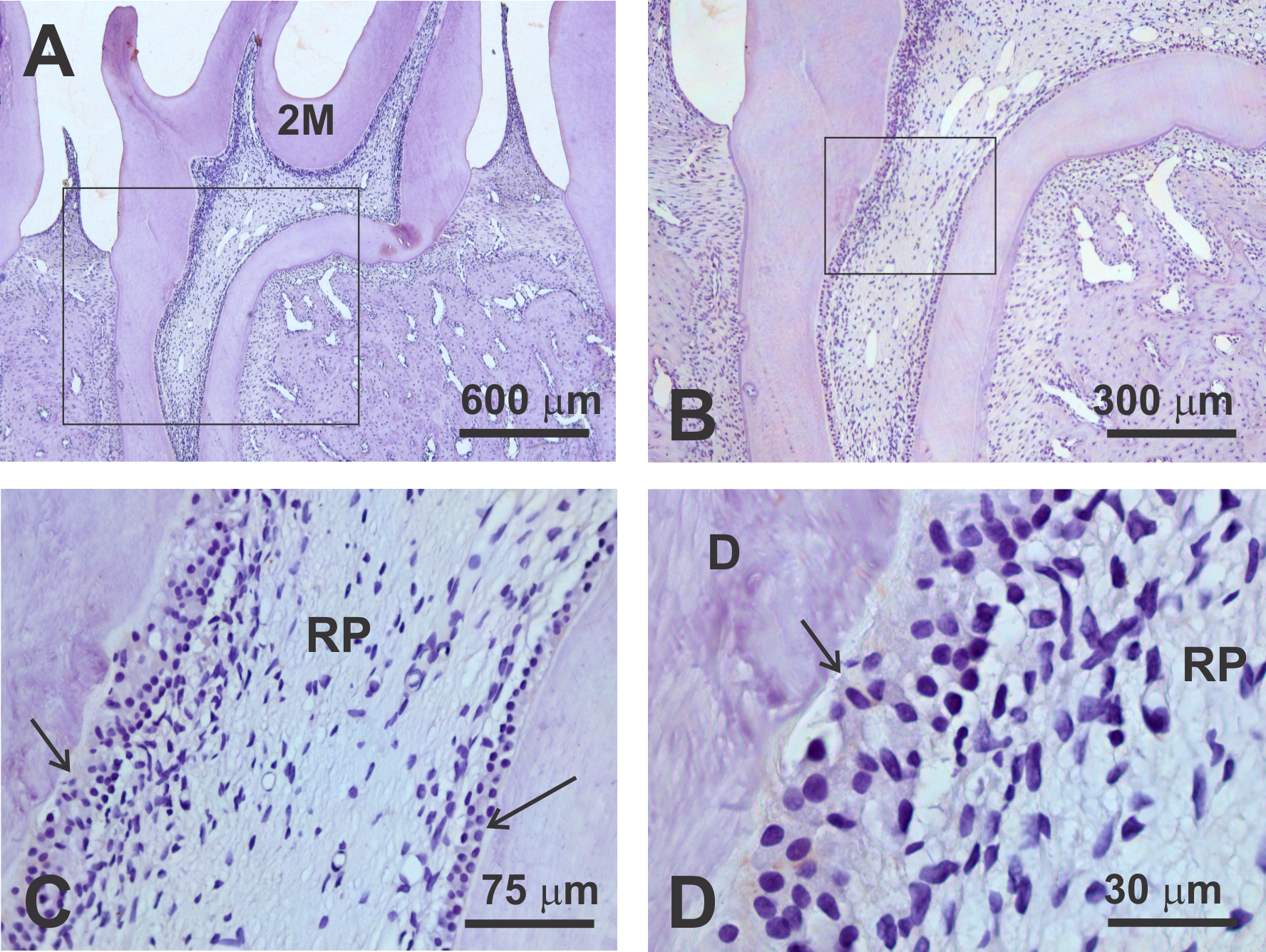

Supplement: S1 Fig — Histological section of mature lower molar teeth of rat (A-D) submitted to adsorption test (1:1, Ten-2 peptide/antibody concentrations) and indirect immunoperoxidase method. A-C show sequential resolution of rat second molar tooth evidencing no immunolabeling in odontoblasts (arrows) or in other pulp cells. D shows high magnification of odontoblast layer with absence of immunolabeling (arrows) in radicular pulp. Abbreviations: 2M, lower second molar; D, dentin; RP, radicular pulp. (TIF) [file pone.0184794.s001.tif]

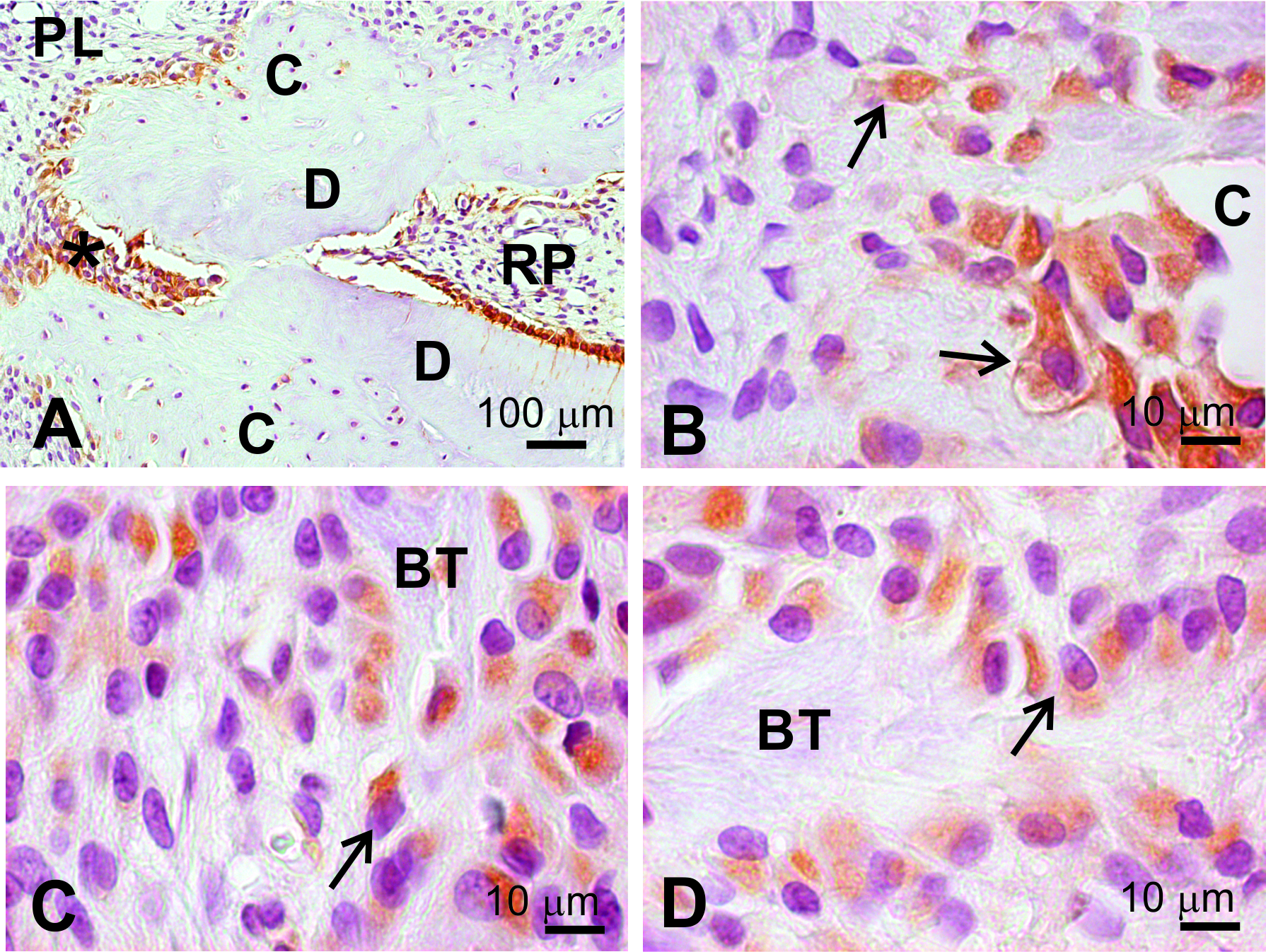

Supplement: S2 Fig — In A-B, presence of cementoblasts (arrows) around the apical foramen (asterisk) exhibiting strong immunolabeling to Ten-2. C-D show some osteoblasts (arrows) situated in the interradicular septum and in the periapical region exhibiting strong Ten-2 immunoreactivity. Abbreviations: BT, bone trabeculae; C, cementum; D, dentin; RP, radicular pulp. (TIF) [file pone.0184794.s002.tif]

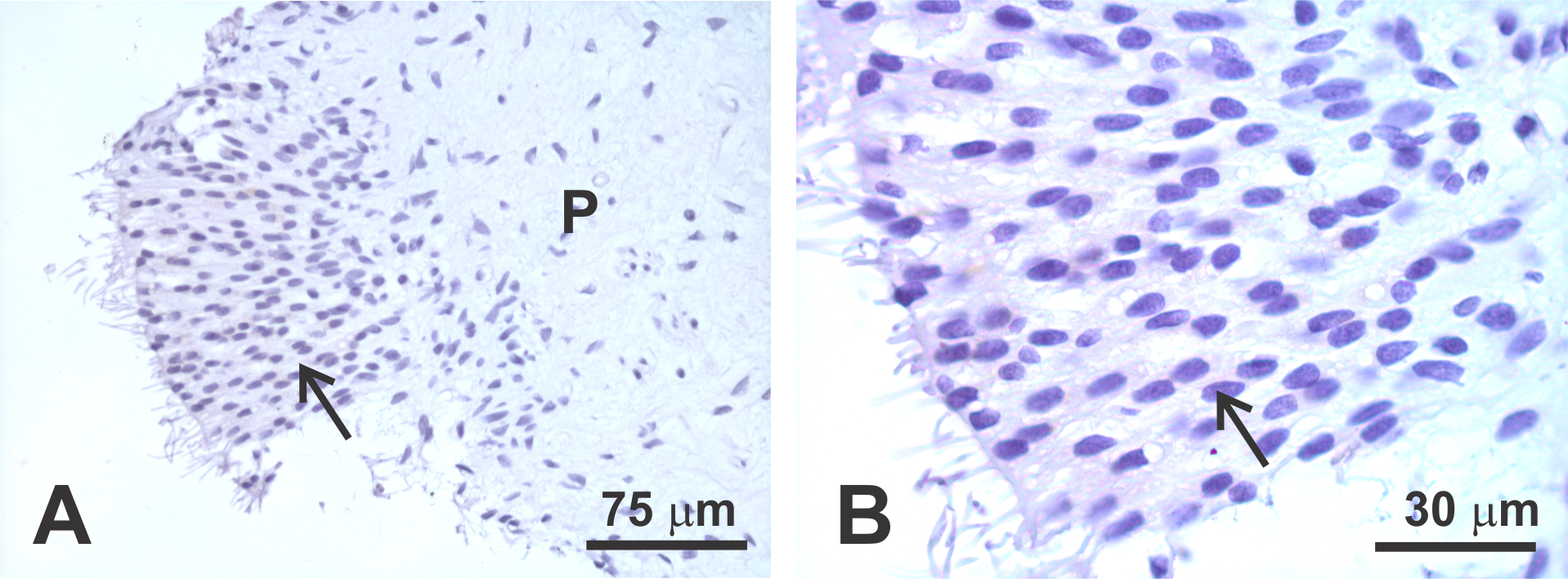

Supplement: S3 Fig — Histological section of mature human dental pulp submitted to adsorption test (1:1, Ten-2 peptide/antibody concentrations) followed by indirect immunoperoxidase method. In A-B, odontoblast layer at high magnification with absence of immunolabelling (arrow). Abbreviation: P, dental pulp. (TIF) [file pone.0184794.s003.tif]
